# HIV-associated non-Hodgkin’s lymphoma- experience from a tertiary referral cancer center

**DOI:** 10.1186/1750-9378-7-S1-P12

**Published:** 2012-04-19

**Authors:** Aruna Alahari Dhir, Sheela P Sawant, Tanuja Shet

**Affiliations:** 1 Department of Medicine, Tata Memorial Hospital, Mumbai, India; 2 Department of Pathology, Tata Memorial Hospital, Mumbai, India

## Background

Infection with human immunodeficiency virus infection (HIV) is associated with an increased risk of Non Hodgkin’s lymphoma (NHL). There is limited data on the treatment and outcome of these lymphomas in India. We describe a retrospective study of 277 HIV infected patients with NHL at a tertiary referral cancer center in Mumbai.

## Material and methods

All patients included in this study were registered at the HIV cancer clinic of the hospital during 2001-2010. All patients were diagnosed to have NHL by tissue biopsy and were confirmed by immunohistochemical tests. Patients were staged with the Ann Arbor staging system. Data of their demographic profiles, immune status, NHL stage, treatment received, response and outcomes were analyzed. We used the gender and age-specific proportion of NHL of the year 2002 that was recorded in the Hospital Cancer Registry to estimate an expected number of NHL among HIV positive cancer patients during the period 2001-2010 (n=770) and the proportional incidence ratio (PIR) was calculated.

## Results

There were 277 patients during the ten year study period. In males the PIR for NHL was 12.6 (95% CI 1.2-14.6) and in females it was 22.1 (95%CI 17.1-28.3) .Among the 277 patients there were 69 females (24.9%) and 208 males (75.1%). The median age of males was 38 years. In females the median age was 37 years.100 Patients (36.1%) were previously known to be HIV positive(range 6 mths-15 years).The CD4 count was less than 200 per cumm in 127/192 (66.14%) patients.76/277 (27.43% ) had current or past history of tuberculosis. 172/277 (62%) patients had extranodal involvement.

168/277 (60.64%) received cancer directed treatment .The data of the 168 patients who received treatment was analyzed. 91/134 (67.91%) had CD4 counts less than 200. 115/168 (68.45%) received antiretroviral therapy. 60% had extranodal involvement.72 (42.9%) had DLCL, 42 (25%) plasmablastic, 21(12.5%) Burkitt’s type and 31 (18.5%) others.90/168 (53.6%) had advanced disease at presentation. All patients were treated with chemotherapy. 54 patients also received RT. The response was evaluated in 96 patients. There was complete response in 46 (47.9%), partial in 15 (15.6%), stable in 6 (6.3%) and 29 (30.2%) patients had progressive disease. The median survival was 25.3 months (range 0-56 months). ART affected survival significantly; however age, sex, CD4 counts at presentation, histopathology, and presence of extranodal involvement and stage of disease did not affect the survival.

## Conclusions

In our study the PIR for NHL was high in HIV-infected patients. The proportion of plasmablastic lymphomas is high. The use of antiretroviral therapy has impacted the overall survival.

